# Dissection of the *E8* locus in two early maturing Canadian soybean populations

**DOI:** 10.3389/fpls.2024.1329065

**Published:** 2024-02-08

**Authors:** Jérôme Gélinas Bélanger, Tanya Rose Copley, Valerio Hoyos-Villegas, Louise O’Donoughue

**Affiliations:** ^1^ Centre de recherche sur les grains (CÉROM) Inc., St-Mathieu-de-Beloeil, QC, Canada; ^2^ Department of Plant Science, McGill University, Montréal, QC, Canada

**Keywords:** soybean, Glycine max, early reproductive traits, genetic linkage map, quantitative trait loci, expression quantitative trait loci, candidate genes

## Abstract

Soybean [*Glycine max* (L.) Merr.] is a short-day crop for which breeders want to expand the cultivation range to more northern agro-environments by introgressing alleles involved in early reproductive traits. To do so, we investigated quantitative trait loci (QTL) and expression quantitative trait loci (eQTL) regions comprised within the *E8* locus, a large undeciphered region (~7.0 Mbp to 44.5 Mbp) associated with early maturity located on chromosome GM04. We used a combination of two mapping algorithms, (i) inclusive composite interval mapping (ICIM) and (ii) genome-wide composite interval mapping (GCIM), to identify major and minor regions in two soybean populations (QS15524_F2:F3_ and QS15544_RIL_) having fixed *E1*, *E2*, *E3*, and *E4* alleles. Using this approach, we identified three main QTL regions with high logarithm of the odds (LODs), phenotypic variation explained (PVE), and additive effects for maturity and pod-filling within the *E8* region: GM04:16,974,874-17,152,230 (*E8-r1*); GM04:35,168,111-37,664,017 (*E8-r2*); and GM04:41,808,599-42,376,237 (*E8-r3*). Using a five-step variant analysis pipeline, we identified *Protein far-red elongated hypocotyl 3* (*Glyma.04G124300*; *E8-r1*), *E1-like-a* (*Glyma.04G156400*; *E8-r2*), *Light-harvesting chlorophyll-protein complex I subunit A4* (*Glyma.04G167900*; *E8-r3*), and *Cycling dof factor 3* (*Glyma.04G168300*; *E8-r3*) as the most promising candidate genes for these regions. A combinatorial eQTL mapping approach identified significant regulatory interactions for 13 expression traits (e-traits), including *Glyma.04G050200* (*Early flowering 3/E6* locus), with the *E8-r3* region. Four other important QTL regions close to or encompassing major flowering genes were also detected on chromosomes GM07, GM08, and GM16. In GM07:5,256,305-5,404,971, a missense polymorphism was detected in the candidate gene *Glyma.07G058200* (*Protein suppressor of PHYA-105*). These findings demonstrate that the locus known as *E8* is regulated by at least three distinct genomic regions, all of which comprise major flowering genes.

## Introduction

Soybean [*Glycine max* (L.) Merr.] is one of the most economically important crops worldwide and is a significant source of vegetable-based protein and oil (Pagano and Miransari, 2016). Domesticated 3,000–9,000 years ago in East Asia from wild soybean (*Glycine soja* Siebold & Zucc.) (Hyten et al., 2006; Lee et al., 2011), the crop has spread throughout the world and is now cultivated in Brazil (36.4%), the United States (34.3%), Argentina (12.1%), China (5.1%), India (3%), Canada (2%), Paraguay (1%), and several other countries (6%) (The American Soybean Association, 2023). While domesticated in a region located between 30°N–45°N and encompassing the eastern Huanghe (Yellow River) basin in North China, South Korea, and Japan (Hyten et al., 2006; Lee et al., 2011), the plant’s ability to adapt to very northern environments is still limited by its short-day photoperiod requirements. Indeed, its recent expansion into northern agricultural regions has only been possible due to major breeding efforts focused on selecting non-photosensitive lines (Zhang et al., 2017). In Canada, the cultivar maturity requirements range from MG000 to MGIII, depending on the region, with an approximate 10-day difference between each group (Bagg et al., 2009). Recently, a putative new maturity group (MG0000) hailing from northeast China and far east Russia has been proposed (Jia et al., 2014; Jiang et al., 2019). This new maturity group demonstrates that expanding soybean’s growing zone beyond its actual northern limits (~54°N) is still possible. However, to unlock soybean’s northern potential, breeders still need to identify novel genes involved in the regulation of early flowering and maturity.

Over the years, several major genes and quantitative trait loci (QTL) involved in reproductive traits, such as *E1*-*E11, J*, *Time of flowering* (*Tof*) *5/11/12/16/18* and *Flowering locus T* (*GmFT*) homologs, have been identified and characterized using forward and reverse genetic approaches (Lin et al., 2020; Gupta et al., 2022). Maturity loci *E1* (*Glyma.06G207800*), *E2* (*Glyma.10G221500*), *E3* (*Glyma.19G224200*), and *E4* (*Glyma.20G090000*) are frequently reported as the most critical players in terms of influence on the final maturity phenotype, explaining more than 60% of the variation in the observed flowering with the proper haplotype combinations (Tsubokura et al., 2014). In addition, determinate habit genes *Dt1* and *Dt2* have been demonstrated to play a complementary role by regulating the growth habit, flowering time, and maturity in soybean (Liu et al., 2010; Ping et al., 2014; Zhu et al., 2023). Loss-of-function variants in *E1*–*E4* contribute to photoperiod insensitivity by indirectly repressing the expression of *FT* orthologs such as *GmFT2a* (*Glyma.16G150700*) and *GmFT5a* (*Glyma.16G044100*) (Kong et al., 2010; Thakare et al., 2011; Watanabe et al., 2011). Studies have shown a high correlation between the latitudinal adaptability/photoperiod insensitivity and the number of recessive alleles for these four *E* loci (Jiang et al., 2014). Several other loci, such as *E9* (*Glyma.16G150700*), *E10* (*Glyma.08G363100*), and *Tof 5/11/12/16/18*, are slowly being implemented in early maturity breeding programs although their effects on flowering and maturity are generally less pronounced than for *E1*–*E4* (Kong et al., 2014; Samanfar et al., 2017; Lu et al., 2020; Dong et al., 2021; Dong et al., 2022; Kou et al., 2022).

The *E8* locus is an interesting locus for breeders as its recessive allele (*e8e8*) imparts a flowering date that is ~5–8 days earlier than its dominant form (Cober et al., 2010). This locus has been mapped between markers Sat_404 and Satt136 on chromosome GM04 (Cober et al., 2010). Two recent research articles have mapped QTL regions located on GM04 which could be *E8* (Kong et al., 2018; Wang et al., 2018); however, the regions identified in these papers are broad [GM04:13,212,370–43,843,500 bp for Kong et al. (2018) and GM04:7,166,748–44,508,948 bp for Wang et al., (2018)] and encompass multiple critical flowering genes such as *E1-like-a* (*Glyma.04G156400*; *E1la*) and *E1-like-b* (*Glyma.04G143300*; *E1lb*), two *E1* homologs. Consequently, these large physical locations suggest that multiple regulatory regions might be controlling flowering on chromosome GM04. Using bioinformatic analyses, seven candidate genes have been proposed for *E8* (Sadowski, 2020), all of which are located between GM04:9,337,214 and GM04:22,755,516.

Through our experiments, we observed significant differences in maturity time between Canadian lines from two early maturing populations (MG00-MG000) that were selected and fixed for identical *E1-E4* alleles, thus suggesting potential novel sources of regulation for these traits. These populations were developed to reduce the background noise generated by *E1-E4* due to their important role in maturity in terms of phenotypic variation. The narrow genetic diversity of Canadian soybean lines, especially within early maturing accessions, suggests that only a handful of regions and causal variants might be contributing to these observed phenotypes (Grainger and Rajcan, 2014). With this study, we aimed to (i) develop a combinatorial QTL analysis approach to map the regions regulating several reproductive traits under field (fluctuating photoperiod with long days during the flowering period) and greenhouse conditions (constant short days) in two plant populations; (ii) perform expression quantitative trait loci (eQTL) analyses to identify interactions with important flowering genes; and (iii) propose candidate genes involved in early maturity in relation to their gene expression level, gene ontology (GO) annotations and/or genetic polymorphism profile.

## Materials and methods

### Plant materials

The full mapping population of 176 F_2:3_ individuals of the QS15524 population (herein named QS15524_F2:F3_) was derived from a single biparental cross between “OAC Vision” ♀ (PI 567787; MG000, earlier maturing accession) × “Maple Arrow” ♂ (PI 548593; MG00, later maturing accession). The full mapping population of 162 F_5:8_ individuals of the QS15544 population (recombinant inbred lines; herein named QS15544_RIL_) was derived from a single biparental cross between “9004” ♀ (PI 592534, US PVP No. 9600050; MG000, earlier maturing accession) × “AAC Mandor” ♂ (MG00, later maturing accession). The “AAC Mandor” parental line is a food-grade soybean cultivar developed by Dr. Elroy Cober at the Ottawa Research and Development Centre of Agriculture and Agri-food Canada (ONT, Canada) after Ottawa Research and Development Centre of Agriculture and Agri-food Canada. Both populations used in this study were developed at the Centre de recherche sur les grains (CÉROM) inc. in Saint-Mathieu-de-Beloeil (QC, Canada). To generate the QS15544_RIL_ population, the offspring of the “9004” × “AAC Mandor” cross were mass multiplied until reaching the F_5_ generation at which point 200 plants were randomly selected and grown over one season in the greenhouse and three seasons in the field for phenotyping. To identify novel QTL, we genotyped each parent to confirm that those were fixed for *E1* (*Glyma.06G207800*) (Xia et al., 2012), *E2* (*Glyma.10G221500*) (Watanabe et al., 2011), *E3* (*Glyma.19G224200*) (Watanabe et al., 2009; Harada et al., 2011; Xu et al., 2013) and *E4* (*Glyma.20G090000*) (Liu et al., 2008; Tsubokura et al., 2013; Tardivel et al., 2019) genes. As such, the genotypes for the “OAC Vision” and “Maple Arrow” parental lines were identified as *e1-nl*/*e2-ns*/*E3Ha*/*e4-SORE-1* for the QS15524_F2:F3_ population. For the QS15544_RIL_ population, the genotypes were *e1-as*/*e2-ns*/*e3-tr*/*e4p.T832QfsX21* for the “9004” and “AAC Mandor” parental lines.

### Growing conditions, tissue sampling, and phenotyping

For the eQTL analyses, the QS15524_F2:F3_ and QS15544_RIL_ populations were grown following a Modified Augmented Design (Lin and Poushinsky, 1983; Lin and Poushinsky, 1985) that was slightly adjusted for greenhouse conditions such that each table contained one parent and 19 individuals. Under these conditions, each population was phenotyped for the number of days to maturity during the winter 2017–2018 (F_2_ generation of the QS15524_F2:F3_ population) and winter 2019–2020 (F_5_ generation of the QS15544_RIL_ population), respectively. For the greenhouse experiments, the plants were sown in one-gallon pots containing a ProMix-garden soil (1:1 v:v) (Premier Tech Horticulture, Rivière-du-Loup, QC, Canada) potting mix, with one seed per pot for the QS15524_F2:F3_ population or three seeds for the QS15544_RIL_ population. Seeds were sown at a depth of 4 cm and inoculated with 1 × 10^8^ colony-forming units of liquid Cell-tech^®^ (Novozymes BioAg, Saskatoon, SK, Canada) *Bradyrhizobium japonicum* at sowing and placed in a greenhouse with the following growing conditions: 12:12, light:dark (L:D), 27°C/24°C (L:D), and 80% relative humidity (Fehr, 1980). Plants were watered daily with a drip irrigation system with increasing volume to meet the plant needs and fertilized weekly alternating with a 15-30-15 or 20-20-20 (nitrogen-phosphorus-potassium) nutrient solution. Five pots of each parent were sown at the same time as the mapping population for a total of 190 study plants for each population. Pots were placed randomly across ten greenhouse tables with 20 pots per table. Due to extremely late maturity, or plant damage, a total of 184 and 182 individuals were retained for the eQTL and QTL analyses for the QS15524_F2:F3_ and QS15544_RIL_ populations, respectively. Leaf tissue was harvested from plants grown in the greenhouse 25 days after sowing (V4 stage) for both populations (McWilliams et al., 2004). Samples were taken four hours after sunrise for RNA extraction, while samples for DNA extraction were taken later in the day. All samples were immediately frozen in liquid nitrogen after harvesting and stored at −80°C until further use. These time points were taken from previously published data indicating highest expression of flowering genes four hours after sunrise (Kong et al., 2010; Sun et al., 2011), while the V4 stage was determined as the optimal stage according to qRT-PCR analyses of the expression of the flowering genes *Glyma.16G150700* (*GmFT2a*) and *Glyma.16G044100* (*GmFT5a*) in the parents.

For the field phenotypes, the QS15524_F2:F3_ and QS15544_RIL_ populations were grown in Saint-Mathieu-de-Beloeil (QC, Canada) using a Modified Augmented Design (Lin and Poushinsky, 1983; Lin and Poushinsky, 1985). The F_3_ generation of the QS15524_F2:F3_ population was grown in single-row plots over two seasons (summers of 2018 and 2021) and the F_6_:F_8_ generations of the QS15544_RIL_ population were grown over the summers of 2020 (one-row plots), 2021 (two-row plots), and 2022 (two-row plots), respectively. The phenotyping of the field traits was performed on 10 plants of the F_3_ generations for the QS15524_F2:F3_. The field phenotypes were recorded as follows: (i) number of days to flowering, as the day of planting to the day at which 75% of the genotype was flowering; (ii) number of days to maturity, as the day of planting to the day at which 95% of the pods within the genotype were at physiological maturity; and (iii) number of days to filling, as the number of days from flowering to maturity. To map the QTL regions for the F_3_ generation of the QS15524_F2:F3_ population, we averaged the observed phenotypes and used them as if those were phenotypes from the F_2_ generation in the mapping pipeline. Phenotypic data distribution, Q-Q plots and Pearson correlation coefficients (PCC) were generated using R version 4.0.4 (R Core Team, 2010). Statistical analyses for the Modified Augmented Design were performed in Agrobase Generation II^®^ (Agronomix Software Inc., 2009). Descriptive statistics (i.e., variance, standard error, kurtosis, and skewness) for the four reproductive phenotypes were calculated using QTL IciMapping 4.2 (Meng et al., 2015). The broad-sense heritability values were estimated using a linear mixed model with the est_herit function implemented in R/qtl2 (Broman et al., 2019). The kinship matrices used to estimate the heritability values were generated with the calc_kinship function implemented in the same package. Statistical differences between the years of data collection were calculated using a paired Student’s t-test (t-test function in R) or a one-way analysis of variance (ANOVA) (aov function in R) using a threshold *p*-value of 0.01.

### Nucleic acid extraction and sequencing

Total DNA was extracted from tissue using the Omega Bio-Tek Mag-bind Plant Kit (Omega Bio-tek, Norcross, GA, USA) with further purification using the Mag-Bind Total Pure NGS (Omega Bio-tek, Norcross, GA, USA). Sampling of the QS15524_F2:F3_ parental lines for the whole genome sequencing (WGS) was performed by pooling the samples from the five pots used to grow each of the parental line, extracting total DNA, and having the libraries prepared at the Génome Québec Innovation Centre (Montréal, QC, Canada) using the NxSeq® AmpFREE Library Preparation kit (Lucigen, Middleton, WI, USA). To do so, the two parental libraries were barcoded, combined and sequenced to a depth of 15X on the Illumina HiSeq X platform with 150 base pair paired-end reads. The WGS data for the QS15524_F2:F3_ and QS15544_RIL_ parental lines were also retrieved from the GmHapMap as available and detailed in Torkamaneh et al. (2020). Phasing of the single-nucleotide polymorphisms (SNPs) data for the QS15524_F2:F3_ population was performed using the resequenced WGS data, whereas the identification of the candidate SNP was performed using the GmHapMap WGS datasets.

To generate the genotyping-by-sequencing (GBS) datasets of the QS15524_F2:F3_ (F_2_ generation) and QS15544_RIL_ (F_5_ generation) mapping populations, the libraries were prepared at the Institute of Integrative Biology and Systems (Laval University, Quebec City, QC, Canada) using the *PstI/MspI* enzymes as detailed in Abed et al. (2019). Samples were randomly divided into two sets of 91 individuals, which were barcoded and pooled to form two libraries per population. Sequencing of the QS15524_F2:F3_ GBS libraries was done by combining a total of 91 barcoded samples per library. Sequencing of each library was done on four Ion PI V3 Chips per library with sequencing performed on the Ion Proton Sequencer and HiQ chemistry at the Institute of Integrative Biology and Systems, for a total of eight sequenced chips. For the QS15544_RIL_ population, samples were randomly divided into two sets of 91 samples and sequenced using the same technologies, with two chips per library.

Total RNA was extracted from samples using a standard Trizol™ (Invitrogen, Waltham, MA, USA) RNA extraction procedure as detailed in the company’s protocol, with two additional ethanol rinses to improve purity. Isolation of messenger RNA (mRNA) was performed using the NEBNext mRNA stranded library preparation kit (New England Biolabs, Ipswich, MA, USA) at the Génome Québec Innovation Centre. A total of 96 samples were barcoded and pooled per final library with one population per library. Each of the libraries was sequenced on two Illumina NovaSeq6000 lanes using S2 or S4 flow cells with 100 base pair paired-end sequencing at the Génome Québec Innovation Centre, for a total of four sequencing lanes. Genome coverage was evaluated to be ≈43.9 M paired-end reads per sample for the QS15524_F2:F3_ and ≈50M reads per sample for the QS15544_RIL_ population.

### Bioinformatics

All sequencing alignment was done using version 2 of the *Glycine max* reference genome (Gmax_275_v2.0). Whole genome sequencing data were processed using the fast-WGS pipeline (Torkamaneh et al., 2018) for the QS15524_F2:F3_ parental lines. Briefly, raw data were aligned to the genome using Burrows-Wheeler Alignment (Li and Durbin, 2009) with the command: bwa mem refGenome Input. Variants were called using Platypus version 0.8.1 (Rimmer et al., 2014) with the following commands: –minReads=2, –minMapQual=20, and –minBaseQual=20. GBS data were processed using the fast-GBS pipeline (Torkamaneh et al., 2017). Briefly, samples were demultiplexed using Sabre version 1.00 (Joshi, 2011), and their adapters removed using Cutadapt (Martin, 2011). The samples were subsequently aligned to the reference genome using Burrows-Wheeler Alignment with the command: bwa mem refGenome Input. Quality checks on the raw data were performed using FastQC software version 0.11.9 (Andrews, 2010). Variants were then called using Platypus version 0.8.1 with the following commands: –minReads=2, –minMapQual=20, and -minBaseQual=20. Genotypes were filtered using vcftools version 0.1.16 (Danecek et al., 2011) to (i) maintain only biallelic sites, (ii) remove InDels, (iii) keep polymorphisms located only on chromosomes and not scaffolds, and (iv) filter allele frequency and count with the –maxmissing 0.2, –maf 0.3, and –mac 4 commands. For the QS15544_RIL_ population only, each SNP and offspring was then filtered based on their heterozygosity using an interquartile range approach {[*Q*_1_ *−k*(*Q*_3_ *−Q*_1_), *Q*_3_ *+k*(*Q*_3_ *−Q*_1_)], *k* = 3, as per Tukey (1977). As such, loci with >14.85% heterozygous calls and offspring with >18.57% heterozygous calls were considered outliers and removed. Missing genotypes for the QS15524_F2:F3_ and QS15544_RIL_ populations were then self-imputed using Beagle version 4.1.0 with 12 iterations (Browning and Browning, 2016). Genotypes were phased with Convert2map https://bitbucket.org/jerlar73/convert-genotypes-to-mapping-files/src/master/ (accessed 12 December 2023) using the parental data from the GmHapMap for the QS15544_RIL_ population and the fast-WGS resequenced data for the QS15524_F2:F3_ parental lines. Subsequently, correction of the genotype calls for the QS15524_F2:F3_ population was performed using Genotype Corrector (Miao et al., 2018) with the software default options (sliding window size of 11 and error rates for homo1 and homo2 of 0.03 and 0.01, respectively) and all the implemented quality checks. For the QS15544_RIL_ population, the removal of the double crossovers was performed using Convert2map. Finally, all genotypes with >10% heterozygous calls were removed from the QS15544_RIL_ dataset before binning with QTL IciMapping version 4.2. For the QS15524_F2:F3_ population, binning was performed with the binning option implemented in Genotype Corrector.

RNA dataset processing was performed using an in-house script comprising multiple publicly available software tools. Briefly, adapters were removed using Trimmomatic version 0.33 (Bolger et al., 2014) with the following options: ILLUMINACLIP:$prog/Trimmomatic-0.33/adapters/TruSeq3-SE.fa:2:30:15\, LEADING:3\ and TRAILING:3\, SLIDINGWINDOW:3:20\ and MINLEN:32\. Filtered reads were then aligned to the soybean reference genome using TopHat2 version 2.1.1 (Kim et al., 2013).

### Map construction and QTL analysis

The genetic linkage maps of the QS15524_F2:F3_ and QS15544_RIL_ populations were generated using QTL IciMapping version 4.2 with the Kosambi mapping function to convert the recombination frequency into centimorgans (cM). The QS15524_F2:F3_ map was generated with “Maple Arrow” as parent A (positive additive effect) and “OAC Vision” as parent B (negative additive effect), whereas the QS15544_RIL_ map was generated with ‘AAC Mandor’ as parent A (positive additive effect) and “9004” as parent B (negative additive effect). In this specific case, a positive additivity relates to the increase in the number of days to flowering, pod-filling, and maturity. The linkage groups were split when gaps exceeded 30 cM and the markers were anchored to their physical positions. The linkage maps with the displayed QTL regions were drawn using the Linkage Map View version 2.1.2 package in R (Ouellette et al., 2018). The condensed versions of the full linkage maps were plotted by https://www.bioinformatics.com.cn/en (accessed 12 December 2022), a free online platform for data analysis and visualization.

For the QTL analyses, we opted for a combinatorial approach using two standard mapping algorithms: ICIM approach implemented in QTL IciMapping version 4.2 (Meng et al., 2015), and Genome-wide compositive interval mapping (GCIM) method in the QTL.gCIMapping.GUI.v2.0.GUI package (Zhang et al., 2020). Briefly, ICIM was performed using the following mapping parameters: (i) deletion of the missing phenotypes; (ii) a scanning interval step of 1 cM and a PIN of 0.001; and (iii) a logarithm of the odds (LODs) threshold determined with 1000 permutations and α of 0.05. GCIM was performed using the fixed model and a walking speed of 1 cM for both populations. In addition, mapping in the QS15524_F2:F3_ population was performed by choosing the restricted maximum likelihood (REML) function implemented in the same software. To remove the minor QTL regions, we subsequently increased the identified ICIM LOD thresholds from 3.99–4.24 (QS15524_F2:F3_) and 3.43–3.57 (QS15544_RIL_) to 5.25, and the GCIM LOD threshold from 2.5 (i.e., the default parameter) to 7.1 (Zhang et al., 2020). Subsequently, we decided to only retain GCIM with a phenotypic variation explained (PVE) ≥3.5% and ICIM regions with a PVE ≥5.5%. Finally, we only retained regions that were either: (i) identified within both populations; (ii) identified by ICIM and GCIM within the same population; or (iii) identified with only one algorithm within only one population but with LOD ≥ 12 and PVE ≥ 20%. For the GCIM regions for which both flanking markers were the same, we considered a ± 100,000 bp region upstream and downstream of the flanking markers when investigating candidate variants. The recombination fraction figures were calculated using the PlotRF function implemented in R/QTL version 1.50 (Broman et al., 2003) and visualized using ASMap version 1.0-4 (Taylor and Butler, 2017) in R. The QTL regions identified with this combinatorial pipeline were named using the following nomenclature: (i) Method (i.e., ICIM or GCIM); (ii) Population (i.e., 24/QS15524_F2:F3_ or 44/QS15544_RIL_); and (iii) QTL trait and associated number (e.g., mat1 for maturity region 1, fill2 for filling region 2, and flow 3 for flowering region 3). To reduce the number of studied regions, we merged the loci that were found in both populations using the following nomenclature: (i) Merg; (ii) chromosome number; and (iii) field (f) or greenhouse conditions (gh). To increase the precision of our QTL mapping procedure, we generated the results both for (i) each year of data and (ii) phenotypic averages for all the studied years. Based on our observations, the results between both types of analysis (i.e., each year and phenotypic averages) were largely comparable for most regions and a preference was given to the phenotypic averages for the main analysis.

### Expression QTL mapping

The mapping of eQTL regions was performed as in Gélinas Bélanger et al. (unpublished). Briefly, eQTL analysis was performed on the DESeq2 normalized transcript abundances of 38,692 genes of the 176 F_2_ lines of the QS15524_F2:F3_ population and 40,218 genes of the 162 F_5_ lines of the QS15544_RIL_ population. Mapping of the eQTL traits was performed using a combinatorial approach that includes the use of three different algorithms: (i) ICIM; (ii) GCIM; and (iii) Interval mapping (IM) from QTL IciMapping version 4.2. The LOD thresholds for ICIM and IM were calculated in QTL IciMapping with 1,000 permutations of 100 sampled expression traits (e-traits) with α of 0.05 and a walking step of 1 cM for genome-wide scanning. Subsequently, global permutation thresholds were calculated as the 95^th^ percentile of the representative null distribution and equaled to (i) 4.01 for ICIM in QS15544_RIL_; (ii) 3.99 for IM in QS15544_RIL_; (iii) 4.13 for ICIM in QS15524_F2:F3_; and (iv) 4.12 for IM in QS15524_F2:F3_. For GCIM, the REML-fixed and fixed model components were respectively chosen for the QS15524_F2:F3_ population and QS15544_RIL_ populations, both with a walking speed of 1 cM. In the QTL.gCIMapping.GUI v2.0 package, the likelihood function is only available to F_2_ populations and was chosen based on prior testing. The GCIM LOD threshold was increased from 2.5 to 7.5 for QS15524_F2:F3_ and 4.0 for QS15544_RIL_ to improve the reliability of the results.

Expression QTL generated by the three algorithms were retained only if they fell within ± 1 Mbp in at least two of the three methods. To do so, the interactions were divided between *trans*-acting and *cis*-acting, and the size of each of the identified eQTL regions (i.e., all of the loci identified with the three aforementioned algorithms) was manually adjusted by adding 500,000 bp both upstream and downstream. The overlapping sets of regions were then identified using the genomic peak Venn function implemented in https://www.bioinformatics.com.cn/en (accessed 12 December 2022), a free online platform for data analysis and visualization. The overlaps were identified using a pairwise comparison (e.g., ICIM vs. IM) using the ICIM interactions as the reference regions in the ICIM versus IM and ICIM versus GCIM analyses. In addition, the IM regions were used as references in the IM vs GCIM analysis. *Trans* interactions overlapping *cis* regions were *de facto* considered as *cis* and excluded from *trans*-interactions hotspot mapping.

### Identification of candidate SNPs and genes

Candidate SNPs and genes were identified using a five-step custom pipeline. First, the prediction of the deleterious effects of the SNPs was performed using Ensembl Variant Effect Predictor (VEP) with Glycine_max_v2.1 (McLaren et al., 2016). Second, putative effects of identified non-synonymous missense polymorphisms were then predicted using Sorting Intolerant From Tolerant 4G (SIFT4g) using “William 82” as the wild-type allele (Ng and Henikoff, 2003; Kumar et al., 2009). To do so, we generated a soybean database using the annotations of *G. max* Wm82.a2.v1 from Ensembl Plants (Yates et al., 2022) and by following the SIFT4G_Create_Genomic_DB guidelines available at https://github.com/pauline-ng/SIFT4G_Create_Genomic_DB (accessed 12 December 2022). The SNPs with SIFT scores <0.05 were classified as putatively deleterious and the ones ≥0.05 were considered as tolerated. Third, we matched the parental genotypes from the GmHapMap (Torkamaneh et al., 2020) dataset with the parental allele causing the additive effect. Fourth, we retained only polymorphisms that were predicted as having moderate or high consequences on the protein structure. Variants located in the 3′ and 5′ (UTR) regions were also retained if those were identified within the sequence of a gene with a validated reproductive function in soybean. Fifth, we generated one custom GO database by retrieving 162 terms flagged as linked to (i) flowering and maturity and (ii) photosynthesis and photoperiodic response from Soybase (Grant et al., 2009) as detailed in Gélinas Bélanger et al. (unpublished). Also, we retrieved 836 soybean genes identified as putatively involved in flowering based on comparative analysis using Arabidopsis orthologs (Zhang et al., 2017). Genes identified as having ≥3 GO annotations, flagged as being an Arabidopsis flowering ortholog, validated for a reproductive function, and/or harboring one or multiple deleterious polymorphisms were prioritized in the downstream analysis.

## Results

### Generation of the populations and phenotypic analysis

**Figure 1:**
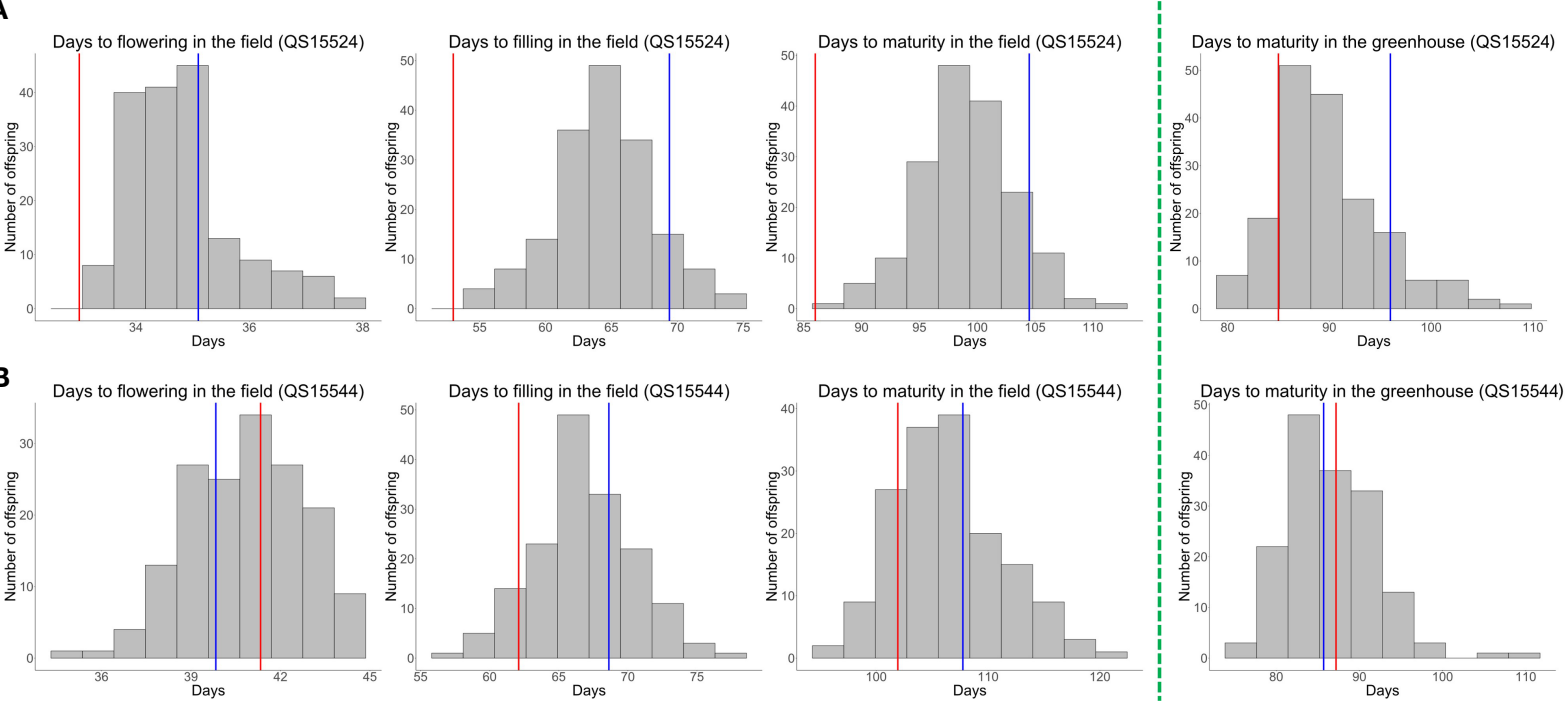
Phenotypic trait data distribution for the QS15524_F2:F3_ and QS15544_RIL_ populations. **(A)** Distribution of the phenotypes for the QS15524_F2:F3_ population in the greenhouse (winter 2017–2018) and in the field (phenotypic average for the summers of 2018 and 2021). Parental lines are indicated with vertical-colored lines. Red lines, “OAC Vision”; blue lines, “Maple Arrow.” **(B)** Distribution of the phenotypes for the QS15544_RIL_ population in the greenhouse (winter 2019-2020) and in the field (phenotypic average for the summers of 2020, 2021 and 2022). Parental lines are indicated with vertical-colored lines. Red lines, “9004”; blue lines, “AAC Mandor.” The green dotted line delineates the field (left-hand side) and the greenhouse (right-hand side) phenotypes.

To perform our experiment, we generated the QS15524_F2:F3_ and QS15544_RIL_ populations and phenotyped both in the greenhouse (one trait; maturity) during the winter and in the field (three traits; flowering, pod-filling, and maturity) during the summer. Both populations exhibited an agronomically important difference in terms of the number of days to flowering in the field, pod-filling in the field, maturity in the field, and maturity in the greenhouse for each year of data (**Supplementary Tables S1A, S1B**) and also their phenotypic average for each trait (**Figures 1A, B**; **Supplementary Tables S1A, S1B**). When comparing both populations, the QS15524_F2:F3_ population always displayed an earlier phenotype for all reproductive traits. Transgressive segregation was mainly observed in the QS15544_RIL_ population based on the respective distribution pattern of the offspring for each trait. The distribution of all of the phenotypes followed a normal distribution, except for the number of days to flowering of the QS15524_F2:F3_ population (**Supplementary Figure S1**; **Tables S1A, S1B**). The broad-sense heritability values for each of the trait and years of data collection were high (i.e., H^2^ ≥ 0.5), except for the number of days to flowering in both populations, thus indicating that genotypes contribute to most of the variation observed in the studied traits (**Supplementary Table S1B**). Likewise, the pairwise PCC for each of the trait and years of data collection were also high (PCC ≥ 0.5), except for the flowering trait (**Supplementary Table S1C**). A significant year effect was detected for all phenotypes based on the t-test and ANOVA analyses (**Supplementary Table S1D**); however, the high-heritability values and PCC between the years suggest that this observation was most likely due to a magnitude effect on the trait. Consequently, the traits were further analyzed using the phenotypic averages for all the studied years.

### Construction of the linkage maps

**Figure 2:**
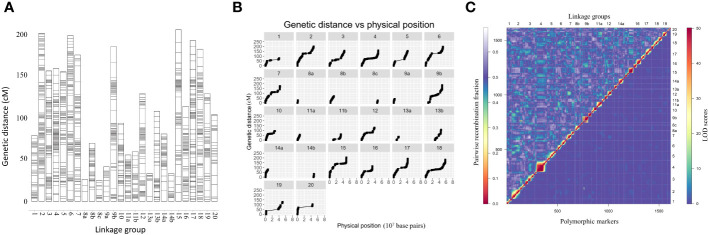
Construction of the linkage map for the QS15524_F2:F3_ population. **(A)** Full linkage map displaying the 26 linkage groups and 1,613 polymorphic markers. **(B)** Plot of the genetic distance vs. the physical position of the markers. **(C)** Pairwise recombination fraction (upper left) and LOD scores for tests of linkage (bottom right) for all 1,613 markers. The upper half represents the recombination fraction between the markers, from the lowest (red color) to the highest (white color). The bottom half displays the LOD score associated with the linkage between each marker pair, from the lowest (blue color) to the highest (red color). Smaller linkage groups have been removed to facilitate visualization.

**Figure 3:**
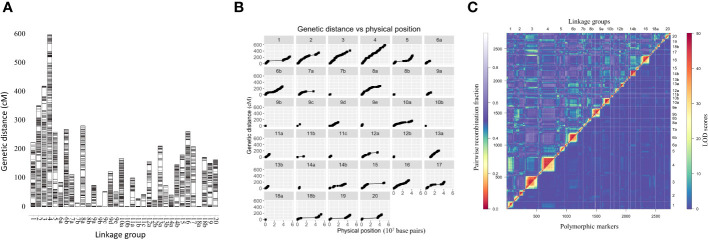
Construction of the linkage map for the QS15544_RIL_ population. **(A)** Full linkage map displaying the 34 linkage groups and 2,746 polymorphic markers. **(B)** Plot of the genetic distance vs. the physical position of the markers. **(C)** Pairwise recombination fraction (upper left) and LOD scores for tests of linkage (bottom right) for all 2,746 markers. The upper half represents the recombination fraction between the markers, from the lowest (red color) to the highest (white color). The bottom half displays the LOD score associated with the linkage between each marker pair, from the lowest (blue color) to the highest (red color). Smaller linkage groups have been removed to facilitate visualization.

Linkage maps based on the segregation of GBS-derived SNP markers for 176 F_2_ lines of the QS15524_F2:F3_ population (**Figure 2A**) and 162 F_5_ lines of the QS15544_RIL_ population were generated (**Figure 3A**). A total of 541,106,451 and 286,844,986 unique single-end reads were generated in the sequencing step for QS15524_F2:F3_ and QS15544_RIL_, respectively. For the final linkage maps, 1,613 (QS15524_F2:F3_; **Supplementary Tables S1E, S1G**) and 2,746 (QS15544_RIL_; **Supplementary Tables S1F, S1G**) polymorphic markers were retained after applying our SNP filtering pipeline. Splitting of the markers distanced by a gap >30 cM resulted in a map with 26 linkage groups with a total length of 2,971 cM, an average genetic distance between the markers of 1.84 cM, and an average length per linkage group of 114.27 cM for QS15524_F2:F3_ (**Table 1**). The same procedure generated 34 linkage groups measuring an average length of 148.77 cM with an average distance between markers of 1.84 cM, and a total length of 5,058 cM in QS15544_RIL_ (**Table 2**). The high quality of both maps was confirmed by plotting the genetic distance versus the physical position (**Figures 2B**, **3B**) and the pairwise recombination fraction and LOD score (**Figures 2C**, **3C**).

**Table 1:** Linkage map characteristics of the QS15524_F2:F3_ population.

Linkage group	Number of markers	LG length (cM)	Average interval (cM)	Linkage group	Number of markers	LG length (cM)	Average interval (cM)
1	47	78.21	1.66	11a	52	54.60	1.05
2	134	200.20	1.49	11b	28	58.63	2.09
3	95	155.42	1.64	12	82	127.97	1.56
4	121	158.38	1.31	13a	11	33.30	3.03
5	86	153.66	1.79	13b	45	107.07	2.38
6	101	197.36	1.95	14a	46	79.91	1.74
7	86	174.26	2.03	14b	16	33.19	2.07
8a	4	26.24	6.56	15	85	204.10	2.40
8b	43	69.38	1.61	16	74	113.13	1.53
8c	12	24.66	2.06	17	100	191.03	1.91
9a	17	40.55	2.39	18	106	181.48	1.71
9b	87	183.60	2.11	19	55	128.08	2.33
10	51	93.34	1.83	20	29	103.29	3.56

**Table 2:** Linkage map characteristics of the QS15544_RIL_ population.

Linkage group	Number of markers	LG length (cM)	Average interval (cM)	Linkage group	Number of markers	LG length (cM)	Average interval (cM)
1	123	224.10	1.82	10b	2	1.32	0.66
2	187	351.10	1.88	11a	50	99.87	2.00
3	228	419.03	1.84	11b	12	27.83	2.32
4	302	596.60	1.98	11c	15	44.36	2.96
5	137	258.57	1.89	12a	77	156.95	2.04
6a	51	84.74	1.66	12b	20	23.10	1.15
6b	181	268.96	1.49	13a	98	210.99	2.15
7a	72	111.90	1.55	13b	31	73.61	2.37
7b	4	14.31	3.58	14a	33	41.34	1.25
8a	130	280.55	2.16	14b	89	145.58	1.64
8b	9	12.31	1.37	15	84	180.38	2.15
9a	55	75.07	1.36	16	167	261.25	1.56
9b	3	3.39	1.13	17	78	208.70	2.68
9c	4	52.69	13.17	18a	4	2.32	0.58
9d	85	122.31	1.44	18b	86	172.01	2.00
9e	29	51.68	1.78	19	72	150.58	2.09
10a	132	168.30	1.28	20	96	162.52	1.69

### Quantitative trait loci mapping

Mapping of the QTL regions was performed using a combinatorial approach with two algorithms (ICIM from the QTL IciMapping software and GCIM from the QTL.gCIMapping R package) for all four traits. The QTL regions were identified both for each year of data (**Supplementary Table S1H**) and the phenotypic averages for all the studied years (presented below). Overall, we identified a total of three regions (*MergGM04f*, *MergGM04gh*, and *MergGM08f*) that were present in both populations (**Table 3**) and also four unique regions that were identified only in QS15544_RIL_ (**Table 4**) using the phenotypic averages. In addition to these major regions, several minor QTL loci were also mapped in both populations (**Supplementary Table S1I**).

**Table 3:** Overlapping quantitative trait loci regions between the QS15524_F2:F3_ and QS15544_RIL_ populations.

Region	Trait	Population	QTL name	Linkage group	Position of the QTL peak (cM)	Confidence interval (cM)		QTL position		LOD	PVE (%)	Additive effect	Dominance effect
						Low	High	Left	Right				
*MergGM04f* (GM04:35,168,111-37,664,017)	Maturity	QS15544_RIL_	*ICIM_44_mat1*	4	400.00	399.50	401.50	GM04:35,168,111	GM04:35,533,929	19.60	22.10	1.81	N/A
	Maturity		*GCIM_44_mat1*	4	400.49	N/A	N/A	GM04:35,533,929	GM04:35,533,929	7.10	8.70	1.79	N/A
	Maturity		*ICIM_44_mat2*	4	418.00	417.50	418.50	GM04:37,662,935	GM04:37,664,017	11.30	10.90	1.27	N/A
	Pod-filling	QS15524_F2:F3_	*ICIM_24_fill1*	4	80.00	79.50	80.50	GM04:36,499,381	GM04:36,941,521	33.80	47.00	3.58	0.05
	Maturity		*ICIM_24_mat1*	4	80.00	79.50	80.50	GM04:36,499,381	GM04:36,941,521	41.40	48.20	3.85	0.26
	Pod-filling		*GCIM_24_fill1*	4	79.88	N/A	N/A	GM04:36,499,381	GM04:36,499,381	46.30	28.00	2.85	0
	Maturity		*GCIM_24_mat2*	4	79.88	N/A	N/A	GM04:36,499,381	GM04:36,499,381	51.60	29.60	3.19	0
*MergGM04gh* (GM04:41,808,599-42,376,237)	Maturity (greenhouse)	QS15544_RIL_	*ICIM_44_matgh1*	4	497.00	495.50	497.50	GM04:42,368,274	GM04:42,376,237	5.80	15.70	2.07	N/A
	Maturity (greenhouse)	QS15524_F2:F3_	*ICIM_24_matgh1*	4	84.00	83.50	84.50	GM04:41,808,599	GM04:42,156,365	12.40	29.40	3.73	-1.19
	Maturity (greenhouse)		*GCIM_24_matgh1*	4	83.57	N/A	N/A	GM04:41,808,599	GM04:41,808,599	11.90	18.70	3.21	0
*MergGM08f* (GM08:47,258,336-47,770,836)	Pod-filling	QS15524_F2:F3_	*ICIM_24_fill2*	8c	14.00	10.50	17.50	GM08:47,258,336	GM08:47,289,756	6.30	5.90	-1.31	-0.10
	Maturity		*ICIM_24_mat4*	8c	14.00	11.50	16.50	GM08:47,258,336	GM08:47,289,756	11.60	8.70	-1.69	0.26
	Pod-filling		*GCIM_24_fill5*	8c	14.00	N/A	N/A	GM08:47,258,336	GM08:47,289,756	13.80	5.20	-1.22	0
	Maturity		*GCIM_24_mat6*	8c	13.65	N/A	N/A	GM08:47,258,336	GM08:47,258,336	13.60	4.40	-1.23	0
	Maturity	QS15544_RIL_	*ICIM_44_mat6*	8b	12.00	11.50	12.00	GM08:47,706,704	GM08:47,770,836	5.40	4.80	0.85	N/A

N/A, not available.

**Table 4:** Unique quantitative trait loci regions identified in the QS15544_RIL_ population.

Region	Trait	QTL name	Linkage group	Position of the QTL peak	Confidence interval (cM)		QTL position		LOD	PVE (%)	Additive effect
				(cM)	Low	High	Left	Right			
GM04:16,974,874-17,152,230	Pod-filling	*ICIM_44_fill2*	4	226.00	225.50	226.50	GM04:16,974,874	GM04:17,152,230	13.21	27.41	1.81
GM07:5,256,305-5,4049,71	Maturity	*ICIM_44_mat5*	7a	17.00	15.50	18.50	GM07:5,256,305	GM07:5,279,354	11.35	11.28	1.30
	Maturity	*GCIM_44_mat5*	7a	22.52	N/A	N/A	GM07:5,404,971	GM07:5,404,971	8.52	3.60	1.15
GM16:5,680,173-5,730,237	Maturity	*GCIM_44_mat2*	16	53.40	N/A	N/A	GM16:5,680,173	GM16:5,730,237	13.07	6.16	-1.51
	Maturity	*ICIM_44_mat3*	16	53.00	52.50	54.50	GM16:5,680,173	GM16:5,730,237	14.67	14.89	-1.55
GM16:22,756,017-23,154,638	Pod-filling	*ICIM_44_fill1*	16	159.00	157.50	161.50	GM16:22,756,017	GM16:23,154,638	5.27	9.65	-1.12
	Pod-filling	*GCIM_44_fill1*	16	159.68	N/A	N/A	GM16:23,154,638	GM16:23,154,638	7.47	13.35	-1.35

N/A, not available.

**Figure 4:**
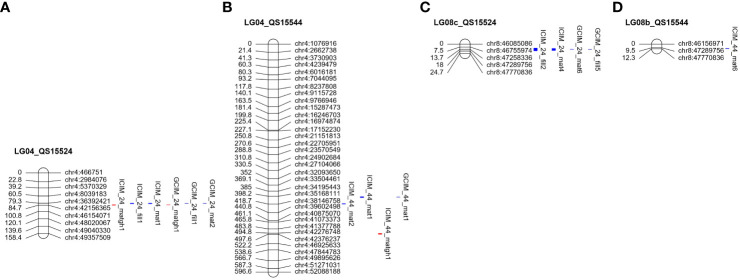
Overlapping quantitative trait loci between the QS15524_F2:F3_ and QS15544_RIL_ populations. Red-marked traits indicate the number of days to maturity in the greenhouse, whereas blue-marked traits are field phenotypes. The QTL regions identified for the QS15524_F2:F3_ **(A)** and QS15544_RIL_ **(B)** populations on chromosome GM04. Two overlapping regions were identified on this chromosome, *MergGM04f* (GM04:35,168,111-37,664,017) and *MergGM04gh* (GM04:41,808,599-42,376,237). A third overlapping region, *MergGM08f* (GM08:47,258,336-47,770,836) was found on chromosome GM08. The identified QTL in this genetic region included populations QS15524_F2:F3_ **(C)** and QS15544_RIL_ **(D)**. The number of markers has been decreased for both chromosomes to facilitate visualization.

For the QS15524_F2:F3_ population, ICIM and GCIM identified a total of 10 QTL on chromosomes GM04 and GM08 (**Table 3**). Overall, the most significant QTL in terms of LOD, PVE, and additive effects were identified on GM04 (**Figure 4A**; **Table 3**). Four QTL were detected in a ≈450 Kbp region located between the GM04:36,499,381 and GM04:36,941,521 flanking markers with ICIM (ICIM_24_fill1 and ICIM_24_mat1) and GCIM (GCIM_24_fill1 and GCIM_24_mat2). These QTL were displaying high LOD (33.80–51.60), PVE (28.00%–48.20%), and additive effects (3.19–3.85 days to maturity; 2.85–3.58 days to pod-filling). For the greenhouse maturity trait, we also identified two QTL for QS15524_F2:F3_ (ICIM_24_matgh1 and GCIM_24_matgh1) that were located between the GM04:41,808,599 and GM04:42,156,365 flanking markers (**Figure 4A**; **Table 3**). These regions were in close physical proximity (± 5 Mbp) to the region encompassing the four field QTL, but those were clearly distinct. For the maturity in the greenhouse QTL, the LOD (11.90 and 12.40), PVE (18.70% and 29.40%), and additive effects (3.2–3.73 difference in the number of days to maturity) were also high, albeit slightly inferior to those observed for the field phenotypes. Four QTL from the field data (ICIM_24_fill2, GCIM_24_fill5, ICIM_24_mat4, and GCIM_24_mat6) were also detected on chromosome GM08 between the GM08:47,258,336 and GM08:47,289,756 flanking markers (**Figure 4C**; **Table 3**). For the four regions located on GM08, the LOD scores were between 6.30 and 13.80 and the PVE between 4.40% and 8.70%.

**Figure 5:**
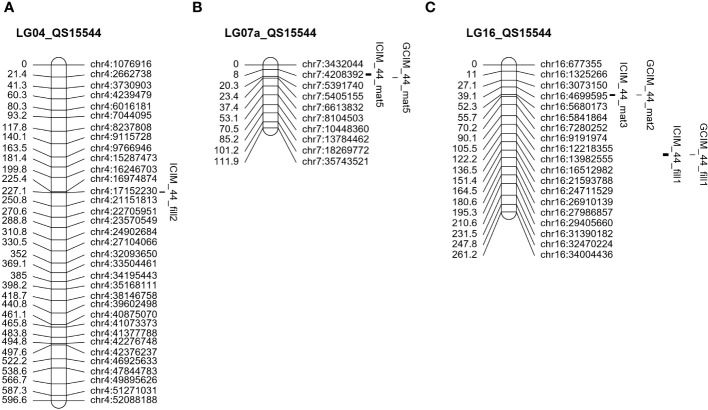
Unique QTL regions identified in the QS15544_RIL_ population. Significant QTL identified on LG04 **(A)**, LG07a **(B)**, and LG16 **(C)**. The number of markers has been decreased on all chromosomes to facilitate visualization.

Using the same approach as for the QS15524_F2:F3_ population, we identified a total of 12 QTL (four with GCIM and eight with ICIM) for the QS15544_RIL_ population (**Tables 3**, **4**). Three QTLs for the number of days to maturity in the field (ICIM_44_mat1, GCIM_44_mat1, and ICIM_44_mat2) were detected on chromosome GM04 in a region comprised between the GM04:35,168,111 and GM04:37,664,017 flanking markers (**Figure 4B**; **Table 3**). The LOD scores for these three traits ranged from 7.10 to 19.60, while the PVE varied between 8.70% and 22.10%. One QTL for the greenhouse maturity trait, ICIM_44_matgh1, with a high additive effect (2.07 days) and PVE (15.70%), was identified between the GM04:42,368,274 and GM04:42,376,237 flanking markers (**Figure 4B**; **Table 3**). Another significant QTL for pod-filling in the field (ICIM_44_fill2), located between the GM04:16,974,874 and GM04:17,152,230 flanking markers, was also identified in the QS15544_RIL_ population, but only with ICIM and not GCIM (**Figure 5A**; **Table 4**). To confirm that this hit was not an artifact of the algorithm, we performed QTL analyses for each season’s data for the pod-filling and maturity traits and also computed their pairwise average for each season’s pair (e.g., 2020 and 2021). A total of nine QTL (ICIM, seven hits; GCIM, two hits) with LOD scores ranging between 6.43 and 20.54 were identified within a ≈2.5 Mbp region starting at GM04:15,748,916 and ending at GM04:18,312,993, thus reinforcing our confidence that this observation was not an artifact (**Supplementary Table S1I**).

The field data also yielded QTL in other regions of the genome. One QTL (ICIM_44_mat6) with a lower LOD score (5.40) and PVE (4.80%) was detected on chromosome GM08 (**Figure 4D**; **Table 3**) in a physically close region (≈500 Kbp) to the one identified in QS15524_F2:F3_. Two QTL, ICIM_44_mat5 and GCIM_44_mat5, with a high-statistical significance (LOD scores of 8.52 and 11.35, respectively) were identified on chromosome GM07 (**Figure 5B**; **Table 4**). Two QTL related to the number of days to maturity, ICIM_44_mat3 and GCIM_44_mat2, were identified on chromosome GM16 between the GM16:5,680,173 and GM16:5,730,237 flanking markers (**Figure 5C**; **Table 4**). In addition, two other QTLs were identified on the same linkage group in a region located between the GM16:22,756,017 and GM16:23,154,638 flanking markers (**Figure 5C**; **Table 4**). All of the QTL identified on GM16 had important LOD, PVE, and additive effects.

### Identification of candidate SNPs and genes

As described in the Material and methods section, we developed a five-step analytical pipeline to discover the best candidate SNPs and genes. This pipeline was subsequently applied to the seven QTL regions identified with ICIM and GCIM (three merged regions and four unique for QS15544_RIL_). For the merged regions, we identified a total of 14 missense polymorphisms (9 SIFT-Tolerated and 5 SIFT-Deleterious), five 3′UTR, and one 5′UTR variant (**Table 5**). For the regions unique to QS15544_RIL_, 10 missense polymorphisms (7 SIFT-Tolerated and 3 SIFT-Deleterious) were identified along with two 3′UTR variants, one splice donor and one stop-gain variant (**Table 6**). Among these polymorphisms, several were located in genes known to be involved in maturity and reproduction. Polymorphisms located in the 3′UTR regions were identified in *E1la* and *Glyma.04G167900* (*Light-harvesting chlorophyll-protein complex I subunit A4; GmLHCA4*) for the merged regions, and in *Glyma.16G044100* (*GmFT5a*) and *Glyma.07G049400* (*Pseudo-response regulator 5d; GmPRR5d*) for the unique regions identified in QS15544_RIL_. The 5′UTR variant was identified in *Glyma.04G166300* (*Pseudo-response regulator 1a; GmPPR1a*). For the *MergGM04gh* region, a SIFT-Tolerated missense polymorphism was detected in *Glyma.04G168300* (*Cycling dof factor 3*; *GmCDF3*), a transcription factor with a known impact on flowering in Arabidopsis. For the unique regions identified in QS15544_RIL_, multiple missense variants were identified in important flowering genes. In the GM04:16,974,874-17,152,230 region, we identified a SIFT-Tolerated missense polymorphism in *Glyma.04G124300* (*Protein far-red elongated hypocotyl 3*; *GmFHY3*) and a SIFT-Deleterious missense polymorphism in *Glyma.04G124600* (*Far1-related sequence 5*; *GmFRS5*). A stop-gain polymorphism was also identified in *Glyma.04G124800* (*Zinc inducted facilitator-like 1*; *GmZIFL1*) in the same region. A SIFT-Tolerated polymorphism was also identified in *Glyma.07G058200* (*Protein suppressor of PHYA-105; GmSPA1*) for the GM07:5,256,305–5,404,971 region. A splice donor variant predicted to have a high impact on the protein structure was identified in *Glyma.16G110700* (*Cytochrome P450*; *GmCYP450*) in the GM16:22,756,017–23,154,638 region.

**Table 5:** Candidate variants for the overlapping quantitative trait loci regions.

Region	Locus, gene	Nucleotide variant		Amino acid variant		Type	Consequence	Gene name	Provider early/shorter phenotype
		Position	W82/MA/OV/MD/90^1^	Position	W82/MA/OV/MD/90^1^				
*MergGM04f* (GM04:35,168,111-37,664,017)	*Glyma.04G156200*	GM04:36,554,187	A/A/G/A/G	1,794	L/L/P/L/P	Missense	Tolerated	*Tetratricopeptide repeat domain protein/Reduced chloroplast coverage 2*	OV & 9004
	*Glyma.04G156400*	GM04:36,758,687	G/G/A/G/A	N/A	N/A	3’UTR	N/A	*E1-like-a*	
	*Glyma.04G156700*	GM04:36,985,491	T/T/A/T/*	270	L/L/Q/L/*	Missense	Tolerated	*Strictosidine synthase-related*	
	*Glyma.04G157000*	GM04:37,049,412	C/C/A/C/A	108	W/W/L/W/L	Missense	Deleterious	*Enolase 1, Chloroplastic*	
	*Glyma.04G157100*	GM04:37,068,001	T/T/G/T/*	352	N/N/J/N/*	Missense	Tolerated	*Pollen-expressed transcription factor 2*	
	*Glyma.04G157100*	GM04:37,069,037	C/C/T/C/T	26	C/C/Y/C/Y	Missense	Deleterious	*Pollen-expressed transcription factor 2*	
*MergGM04gh* (GM04:41,808,599-42,376,237)	*Glyma.04G166300*	GM04:41,757,388	G/G/T/G/*	N/A	N/A	5’UTR	N/A	*Pseudo-response regulator 1a*	OV & 9004
	*Glyma.04G167900*	GM04:42,126,107	T/T/A/T/A	N/A	N/A	3’UTR	N/A	*Light-harvesting chlorophyll-protein complex I subunit A4*	
	*Glyma.04G167900*	GM04:42,126,847	A/A/G/A/G	N/A	N/A	3’UTR	N/A	*Light-harvesting chlorophyll-protein complex I subunit A4*	
	*Glyma.04G167900*	GM04:42,126,965	G/G/A/G/A	N/A	N/A	3’UTR	N/A	*Light-harvesting chlorophyll-protein complex I subunit A4*	
	*Glyma.04G167900*	GM04:42,127,008	G/G/T/G/T	N/A	N/A	3’UTR	N/A	*Light-harvesting chlorophyll-protein complex I subunit A4*	
	*Glyma.04G168300*	GM04:42,192,025	C/C/A/C/A	306	Q/Q/H/Q/H	Missense	Tolerated	*Cycling dof factor 3*	
	*Glyma.04G169200*	GM04:42,358,749	G/G/C/G/C	238	R/R/G/R/G	Missense	Deleterious	*EMB514*	
	*Glyma.04G169200*	GM04:42,359,864	A/A/G/A/G	115	L/L/S/L/S	Missense	Tolerated	*EMB514*	
*MergGM08f* (GM08:47,258,336-47,770,836)	*Glyma.08G362400*	GM08:47,378,990	C/C/T/T/C	867	G/G/E/E/G	Missense	Deleterious	*Clathrin interactor 1*	MA & 9004
	*Glyma.08G366200*	GM08:47,712,475	C/C/T/T/C	237	D/D/N/N/D	Missense	Deleterious	*U11/U12 small nuclear ribonucleoprotein 35 Kda protein*	
	*Glyma.08G366400*	GM08:47,724,957	G/G/A/A/G	115	G/G/S/S/G	Missense	Tolerated	*F1O19.11 protein*	
	*Glyma.08G366400*	GM08:47,725,261	A/A/G/G/A	216	H/H/R/R/H	Missense	Tolerated	*F1O19.11 protein*	
	*Glyma.08G366600*	GM08:47,736,890	A/A/G/G/A	655	D/D/G/G/D	Missense	Tolerated	*Phosphatidylinositol N-acetylglucosaminyltransferase subunit P down syndrome critical region protein 5-related*	
	*Glyma.08G367000*	GM08:47,756,614	C/C/A/A/C	53	A/A/S/S/A	Missense	Tolerated	*UDP-glycosyltransferase 72B2-related*	

^1^ W82, William 82; MA, Maple Arrow; OV, OAC Vision; MD, AAC Mandor; 90, 9004. An asterisk (*) indicates a heterozygote for the SNP of interest.

N/A, not available.

**Table 6:** Candidate variants for the unique quantitative trait loci regions identified in the QS15544_RIL_ population.

Region	Locus, gene	Nucleotide variant		Amino acid variant		Type	Consequence	Gene name	Provider early/shorter phenotype
		Position	W82/MA/OV/MD/90^1^	Position	W82/MA/OV/MD/90^1^				
GM04:16,974,874-17,152,230	*Glyma.04G124300*	GM04:16,097,210	G/G/G/T/G	375	C/C/C/F/C	Missense	Tolerated	*Protein far-red elongated hypocotyl 3*	9004
	*Glyma.04G124600*	GM04:16,331,703	C/C/*/*/T	350	C/C/*/*/Y	Missense	Deleterious	*Far1-related sequence 5*	
	*Glyma.04G124800*	GM04:16,374,159	T/T/T/G/T	188	N/A	Stop-gain	N/A	*Zinc inducted facilitator-like 1*	
GM07:5,256,305-5,404,971	*Glyma.07G049400*	GM07:4,202,517	T/T/T/T/C	N/A	N/A	3’UTR	N/A	*Pseudo-response regulator 5d*	9004
	*Glyma.07G058200*	GM07:5,200,811	C/C/C/C/*	731	R/R/R/R/*	Missense	Tolerated	*Protein suppressor of PHYA-105*	
GM16:5,680,173-5,730,237	*Glyma.16G044100*	GM16:4,136,378	C/A/A/A/C	N/A	N/A	3’UTR	N/A	*Flowering locus T*	MD
	*Glyma.16G050300*	GM16:4,820,456	G/A/*/A/G	122	T/I/*/I/T	Missense	Tolerated	*Fusca3*	
	*Glyma.16G057200*	GM16:5,596,915	T/A/A/A/T	301	N/I/I/I/N	Missense	Tolerated	*Baf60/Chc1*	
	*Glyma.16G057200*	GM16:5,597,545	G/A/A/A/G	117	R/C/C/C/R	Missense	Tolerated	*Baf60/Chc1*	
GM16:22,756,017-23,154,638	*Glyma.16G109600*	GM16:23,645,426	G/G/G/G/C	24	E/E/E/E/D	Missense	Tolerated	*RNA-binding glycine-rich protein D4*	MD
	*Glyma.16G109600*	GM16:23,645,448	G/G/G/G/A	32	A/A/A/A/T	Missense	Deleterious	*RNA-binding glycine-rich protein D4*	
	*Glyma.16G110400*	GM16:24,358,614	A/A/A/A/*	178	K/K/K/K/*	Missense	Tolerated	*Apyrase 7*	
	*Glyma.16G110400*	GM16:24,358,915	T/T/T/T/G	278	H/H/H/H/Q	Missense	Deleterious	*Apyrase 7*	
	*Glyma.16G110700*	GM16:24,403,586	T/T/T/T/C	N/A	N/A	Splice donor variant	N/A	*Cytochrome P450*	

**^1^** W82, William 82; MA, Maple Arrow; OV, OAC Vision; MD, AAC Mandor; 90, 9004. An asterisk (*) indicates a heterozygote for the SNP of interest.

N/A, not available.

### Mapping of eQTL interactions

**Figure 6:**
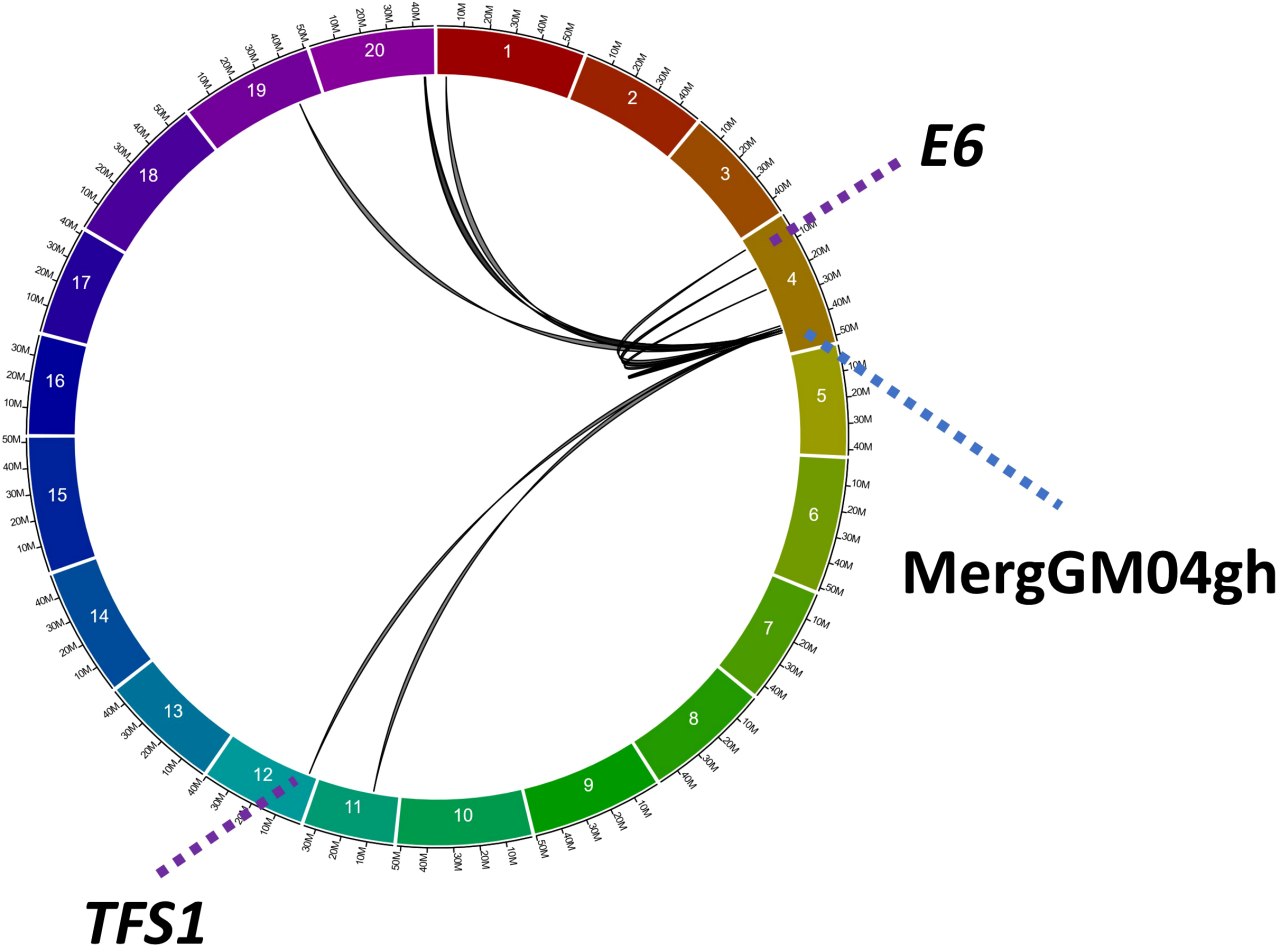
*Trans* and *cis* expression quantitative trait loci for the *MergGM04gh* region. Interactions between this region and 13 different e-traits have been identified using a combination of three algorithms (IM, ICIM, and GCIM). Black lines underline the eQTL interactions between the *MergGM04gh* region and its target genes. Purple dotted lines indicate the positions of two genes involved in flowering: *Glyma.04G050200* (*GmELF3/E6* locus) and *Glyma.12G048500* (*GmTFS1*). Blue dotted line indicates the location of the *MergGM04gh* region.

Using the greenhouse data from the QS15524_F2:F3_ and QS15544_RIL_ populations, we performed a transcriptome-wide eQTL study (Gélinas Bélanger et al., unpublished) using a combinatorial mapping approach with three algorithms (IM, ICIM, and GCIM) designed specifically to identify *cis* and *trans* quantitative e-traits. From these results, we identified several e-traits regulated by the *MergGM04gh* region identified in this present study in the QS15544_RIL_ population. For the QS15544_RIL_ population, we identified a total of 13 e-traits regulated by the *MergGM04gh* region (**Figure 6**; **Supplementary Table S1J**). The e-traits were identified on six chromosomes (GM01, GM04, GM11, GM12, GM19, and GM20) with chromosome GM04 having the highest number of e-traits, seven in total. The *Glyma.04G165400* gene was found to be regulated by *cis* and *trans* interactions from regions located in close physical proximity. Two of the regulated genes were *Glyma.04G050200* (*Early flowering 3/E6* locus; *GmELF3*) and *Glyma.12G048500* (*Target of FLC And SVP1; GmTFS1*), the former being as a light *Zeitnehmer (*“*time-taker*”*)* and thermosensor circadian clock component in Arabidopsis and the latter being an AP2/B3-like transcriptional factor promoting floral transition in Arabidopsis. Both of them were regulated by *trans* interactions. No eQTL interactions were identified for the *MergGM04gh* region in the QS15524_F2:F3_ population.

## Discussion

### Chromosome GM04 is a hub for early reproductive traits

Chromosome GM04 is known to host several major loci (e.g., *E6* and *E8*) and genes (e.g., *E1La* and *E1Lb*) that are involved in the regulation of early reproductive traits (Zhang et al., 2017; Gupta et al., 2022). In addition, this chromosome is known to host a large number of Arabidopsis orthologs (52 genes out of 836) involved in flowering (Zhang et al., 2017). Dissecting QTL regions from this chromosome is challenging due to the close proximity and interplay of several of these orthologous flowering genes, as can be observed in Kong et al. (2018) and Wang et al. (2018) in which the QTL regions encompassed GM04:13,212,370–43,843,500 and GM04:7,166,748-44,508,948, respectively. In this study, we generated high-density GBS-derived linkage maps for chromosome GM04 in two plant populations and performed QTL mapping using a combinatorial approach composed of two mapping algorithms (ICIM and GCIM) with the intent of dissecting the large QTL region normally identified on this chromosome.

In the present study, three distinct loci were identified within the *E8* locus: GM04:16,974,874-17,152,230 (*ICIM_44_fill2*), *MergGM04f*, and *MergGM04gh*. In both populations, the greenhouse (*MergGM04gh*; GM04:41,808,599-42,376,237) and field (*MergGM04f* region; GM04:35,168,111-37,664,017) QTL were identified nearby on the same chromosome. We consider that the *MergGM04gh* and *MergGM04f* regions are distinct due to the large distance separating the regions and the different photoperiod of each growth system (e.g., fluctuating long days in the field vs. constant short days in the greenhouse). Our results demonstrate that *E8* is regulated by three distinct genomic regions on chromosome GM04, which all encompass or are closely located to flowering genes. To dissect *E8* into smaller regions, we decided to split the locus into three distinct regions using the following nomenclature; (i) *E8-r1*, which corresponds to the GM04:16,974,874-17,152,230 identified in QS15544_RIL_ (**Table 4**); (ii) *E8-r2*, which corresponds to the *MergGM04f* (position GM04:35,168,111-37,664,017) identified in both populations (**Table 3**); and (iii) *E8-r3*, which corresponds to the *MergGM04gh* (position GM04:41,808,599-42,376,237) region identified in both populations under greenhouse conditions (**Table 3**). All three regions, listed as ECqMG-4.1 for *E8-r1*, qMG-4.3 for *E8-r2*, and ECqMG-4.4 for *E8-r3*, were previously identified in a genome-wide association study (GWAS) performed with a 16,879 accession panel (Zimmer et al., 2021); however, all of them were only associated with late maturity (MG0 and above) and none with super early maturity (i.e., MG000-MG00) such as the lines used in this study. To the best of our knowledge, this is the first time these alleles are reported for cultivars belonging to the MGs 000 and 00. Additionally, this is the first time these alleles have been demonstrated to have cumulative additive effects to generate an early maturity phenotype. Overall, the high-heritability values for each of the pod-filling and number of days to maturity traits suggest that these QTL could be used in the breeding of early maturing cultivars.

### *E8-r1* locus

The *E8-r1* (GM04:16,974,874-17,152,230) region comprises nine genes and has a high impact on pod-filling (LOD 13.2 and PVE 27.4%), leading to an earlier phenotype by 1.81 (ICIM) days in QS15544_RIL_ (**Table 4**). As previously mentioned, the statistical associations with this region were more challenging to map, with QTL identified starting at GM04:15,748,916 and ending at GM04:18,312,993 with each season’s data and pairwise average for each season’s pair (**Supplementary Table S1I**). None of the nine genes found within the region were previously found to be associated with reproductive phenotypes in soybean or Arabidopsis in the literature; however, we identified two variants, GM04:16,097,210 (*Glyma.04G124300*) and GM04:16,331,703 (*Glyma.04G124600*), located in neighbouring genes that were previously identified as potential candidates for *E8* in Sadowski (2020) (**Table 6**). The GM04:16,097,210 SNP is a G→T SIFT-Tolerated missense polymorphism located at the amino acid position 375 of *Glyma.04G124300*. This polymorphism was found to be present only in “AAC Mandor” and possibly causes a longer pod-filling. The *Glyma.04G124300* gene belongs to the FAR1/FHY3 family which are essential proteins involved in the phytochrome A controlled far-red responses (Lin and Wang, 2004) and positive regulators of chlorophyll biosynthesis (Tang et al., 2012) in Arabidopsis. Furthermore, this family is also involved in the activation of the gene expression of *Circadian clock associated1* (*AtCCA1*) in Arabidopsis which serves as a key component of the core oscillator of the circadian clock (Liu et al., 2020). In Sadowski (2020), *Glyma.04G124300* was considered as a promising candidate, but inferior to *Glyma.04G124600*, another member of the FAR1/FHY3 family. In our variant analysis, *Glyma.04G124600* exhibits a SIFT-Deleterious missense polymorphism C→T on the third exon at amino acid position 350 in “AAC Mandor”; however, “AAC Mandor” is heterozygous for this polymorphism, and more investigation would be required to know if this SNP could be causal. In addition, a T→G stop-gain variant was identified in *Glyma.04G124800*, an ortholog of the Arabidopsis gene *AtZIFL1*. In Arabidopsis, this gene is known to be involved in root development, gravitropism, stomatal movements, and basipetal auxin transport (Remy et al., 2013). Its unconfirmed role in maturity makes *GmZIFL1* less likely to be the regulator at the source of the GM04:16,974,874-17,152,230 region although its polymorphism is predicted to be highly deleterious. On the whole, our results suggest that *Glyma.04G124300* and *Glyma.04G124600* are currently the best candidate genes for the *E8-r1* locus.

### *E8-r2* locus

The *E8-r2* locus (*MergGM04f* region) comprises seven QTL (four in QS15524_F2:F3_ and three in QS15544_RIL_) with important effects on the observed phenotypes, especially those identified for the QS15524_F2:F3_ population (**Table 3**). In the QS15524_F2:F3_ population, the additive effects identified for this region represented an average earlier pod-filling phenotype of 2.85 (GCIM)/3.58 (ICIM) days and an average earlier maturing phenotype of 3.19 (GCIM)/3.85 (ICIM) days for the “OAC Vision” allele. In the QS15544_RIL_ population, this additive effect caused an average earlier maturity of 1.27 (ICIM; GM04:35,168,111-35,533,929 sub-region) and 1.81 (ICIM; GM04:37,662,935-37,664,017 sub-region) days in the offspring having the “9004” allele. It is currently impossible to attest if the QTL observed in the QS15524_F2:F3_ and QS15544_RIL_ populations stem from the same or different regulators; however, based on an analysis of the SNPs identified in the GmHapMap dataset and located within coding regions of genes located within *E8-r2*, a high homology exists within SNPs of the later maturing parental lines (“Maple Arrow” and “AAC Mandor”) versus the earlier maturing parental lines (“OAC Vision” and “9004”) (data not shown). Consequently, this evidence suggests that the causal variants might be the same. To the best of our knowledge, no candidate genes have yet been proposed for this locus, despite being located within the *E8* large-range region and close to a GWAS hit (GM04:38,274,140) identified by Zhang et al. (2015). The narrow *E8-r2* sub-region of the QS15524_F2:F3_ population (GM04:36,499,381-36,941,521) comprises only six genes, including *E11a*, a major transcription factor involved in flowering and maturity that has been validated for the *Tof4* QTL (Liu et al., 2022; Dong et al., 2023) (**Table 5**). Silencing of *E1la* using virus-induced gene silencing upregulates the expression of *GmFT2a* and *GmFT5a*, leading to an earlier flowering (Xu et al., 2015). In our study, a G→A 3′UTR polymorphism was identified at position GM04:36,758,687 in both “OAC Vision” and “9004”, which are the providers of the allele causing an earlier maturity. The 3′UTR region is involved in a plethora of functions, such as RNA stability, translation, and localization, and harbors binding sites for microRNAs and RNA-binding proteins (Steri et al., 2018). In consequence, polymorphisms in a binding site can lead to modifications in the level of gene expression. The presence of *E1la* in the narrow *E8-r2* QS15524_F2:F3_ sub-region of the QS15524_F2:F3_ population and the fact that none of the five other proposed SNPs are located in flowering orthologs suggest that *E1la* is the best candidate for the *E8-r2* region.

### *E8-r3* locus

The *MergGM04gh* region is the only region associated with the number of days to maturity in the greenhouse phenotype and was identified in both populations with ≈200 Kbp separating the QS15524_F2:F3_ QTL from those observed in QS15544_RIL_, suggesting that the causal variant could be the same (**Table 3**). The *MergGM04gh* is related to an earlier maturity phenotype by 3.21 (GCIM)/3.73 (ICIM) days in the QS15524_F2:F3_ population and 2.07 (ICIM) days in the QS15544_RIL_ population under constant short days. Based on our QTL analysis, this earlier flowering phenotype is provided by ‘OAC Vision’ and “9004” in QS15524_F2:F3_ and QS15544_RIL_, respectively. Overlapping or closely located biparental and GWAS QTL have been previously identified by Wang et al. (2015); Sun et al. (2013); Zhang et al. (2015); Mao et al. (2017), and Liu et al. (2021), with several candidate genes being proposed. In our study, the *MergGM04gh* region comprises 28 genes, including two candidate genes with polymorphisms of interest: *Glyma.04g168300* (*GmCDF3*) and *Glyma.04G167900* (*GmLHCA4*) (**Table 5**). Another gene of interest, *Glyma.04G166300* (*GmPRR1a*), is located at ≈50 Kbp upstream of the region. *Glyma.04G168300* (*GmCDF3*) is a Dof-type zinc finger domain-containing transcription factor that was suggested as a candidate maturity gene by Mao et al. (2017). Corrales et al. (2017) recently demonstrated that *AtCDF3* overexpression promotes late flowering partly by controlling the expression of the CBF/DREB2A-CRT/DRE and ZAT10/12 modules in the Columbia (Col-0) ecotype. To the best of our knowledge, its impact on soybean flowering has not been validated yet. In our study, a C→A missense SIFT-Tolerated missense polymorphism has been identified at amino acid position 306 in *Glyma.04G168300*/*GmCDF3*. Based on our analysis of the variants, “OAC Vision” and “9004” exhibit the same genotypes for this polymorphism, supporting it as a potential candidate gene for this region. Additionally, we detected four SNPs (positions GM04:42,126,107, GM04:42,126,847, GM04:42,126,965, and GM04:42,127,008) located in the 3’UTR region of *Glyma.04G167900* (*GmLHCA4*). Overall, these four variants all display the same genotype pattern, with “OAC Vision” and “9004” being the providers of the early flowering alleles. Liu et al. (2021) investigated the role of *Glyma.04G167900* (*GmLHCA4*) and observed a 1.8-day difference in the number of days to flowering between two *GmLHCA4* haplotypes. The PSEUDO RESPONSE REGULATOR (PRR) family regulates many biological processes in Arabidopsis, including photoperiodic flowering, growth, stress response, and regulation of the circadian clock (Hayama et al., 2017; Li et al., 2019; Kim et al., 2022), with several homologs found within the soybean genome. The domestication of the *Glyma.12G073900* (*GmPRR3b*) gene in soybean has been associated with an early flowering phenotype due to the presence of a causal SNP at position GM12:5,520,945 (Li et al., 2020). In our study, we identified a G→T polymorphism in the 5’UTR region at position GM04:41,757,388 of the *Glyma.04G166300* (*GmPRR1a*) gene that is present in “OAC Vision” and “9004” (heterozygous). On the whole, our results suggest that *Glyma.04G168300* (*GmCDF3*), *Glyma.04G167900* (*GmLHCA4*), and *Glyma.04G166300* (*GmPRR1a*) are the best candidates for *E8-r3*.

### Unique QTL in the QS15544_RIL_ population

Using our combinatorial approach, we detected four additional QTL regions (i.e., GM07:5,256,305-5,404,971; GM16:5,680,173-5,730,237; and GM16:22,756,017-23,154,638) that were identified only in the QS15544_RIL_ population, possibly due to a higher number of recombination events and a greater statistical power due to the decreased number of heterozygotes in comparison to QS15524_F2:F3_. Following the identification of these unique regions, those were narrowed to a total of 11 candidates with our five-step variant calling pipeline. For the GM07:5,256,305-5,404,971 region, we identified that the inbred lines carrying the “9004” allele mature between 1.15 (GCIM) and 1.30 (ICIM) days earlier than those harboring the “AAC Mandor” allele. This region was previously identified by Wang et al. (2004) with the Satt567 (position GM07:4,559,602) and Satt463 (position GM07:8,283,465) markers, with four QTL reported in Soybase (i.e., Pod maturity 14-4, First flower 6-1, Pod maturity 10-2 and Reproductive stage length 4-3). Cheng et al. (2011) also identified a QTL between Satt540 (position GM04:5,010,696) and Satt435 (Soybase biparental QTL Reproductive stage length 5-4). For the GM07:5,256,305-5,404,971 region, we identified a SIFT-Tolerated missense polymorphism at position GM07:5,200,811 of the *Glyma.07G058200 GmSPA1* gene. Han et al. (2021) identified a GWAS QTL at position GM7:5,059,730 for the number of days to flowering in soybean and proposed *GmSPA1* as the best candidate for this hit. In Arabidopsis, *AtSPA1* is a WD (tryptophan–aspartic acid)–repeat protein involved in the regulation of the circadian clock and photomorphogenesis in a light-responsive repressor manner (Hoecker et al., 1999; Yang et al., 2005).

The GM16:5,680,173-5,730,237 region has an impact on the number of days to maturity of the QS15544_RIL_ population, with the offspring harboring the “AAC Mandor” allele reaching maturity 1.51 (GCIM)/1.55 (ICIM) days before the ones harboring the “9004” allele. This region lies close (~1.5 Mbp) to *Glyma.16G044100* (*GmFT5a*) and *Glyma.16G044200* (*GmFT3a*), two major homologs involved in flowering and maturity (Liu et al., 2018; Lee et al., 2021). The region is close to the GWAS QTL First Flower 4-g63 (position GM16:5,799,540) (Mao et al., 2017). No gene has been proposed by Mao et al. (2017) for this region. Using our pipeline, we did not find any promising variants within the region; however, four putatively deleterious SNPs were identified upstream of the region in *Glyma.16G044100* (*GmFT5a*), *Glyma.16G050300* (*Fusca3*; *GmFUS3*), and *Glyma.16G057200* (*Baf60*; *GmBAF60*).

## Conclusion

In conclusion, the QS15524_F2:F3_ and QS15544_RIL_ plant populations were generated using fixed alleles for *E1*–*E4*, which enabled us to identify overlapping regions and unique QTL regions involved in reproductive traits. Our results demonstrate that the major *E8* locus is composed of three separate regions (*E8-r1, E8-r2*, and *E8-r3*) with major additive effects. In addition, we demonstrate that eQTL interactions with the major flowering gene *GmELF3*/*E6* and 12 other e-traits stem from regions located within *E8-r3* or nearby. Several other unique QTL regions regulating reproductive traits were also identified in QS15544_RIL_ on chromosomes GM07, GM08, and GM16. With our five-step variant calling pipeline, we were able to identify candidate SNPs and genes located within or near all of the identified QTL regions. Altogether, our results demonstrate that novel major genes controlling early maturity can still be identified and incorporated into early maturing material. Nonetheless, in-depth functional characterization of these candidate genes remains necessary to confirm their role in early pod-filling and maturity.

## Data availability statement

The original contributions presented in the study are publicly available. This data can be found here: PRJNA1035514.

## Author contributions

JG: Conceptualization, Data curation, Formal analysis, Investigation, Methodology, Visualization, Writing – original draft, Software. TC: Conceptualization, Methodology, Supervision, Validation, Writing – review & editing, Data curation, Investigation, Software. VH-V: Supervision, Writing – review & editing. LO’D: Conceptualization, Funding acquisition, Project administration, Resources, Supervision, Validation, Writing – review & editing, Investigation.

## Supplementary material

Supplementary Table S1 Quantile–quantile (Q-Q) plots of phenotypic traits.
