# Supplementary figures and images for: Dissection of the E8 locus in two early maturing Canadian soybean populations
Source: Front Plant Sci. 2024 Feb 8;15:1329065. doi: 10.3389/fpls.2024.1329065 (PMC10881665; doi:10.3389/fpls.2024.1329065)

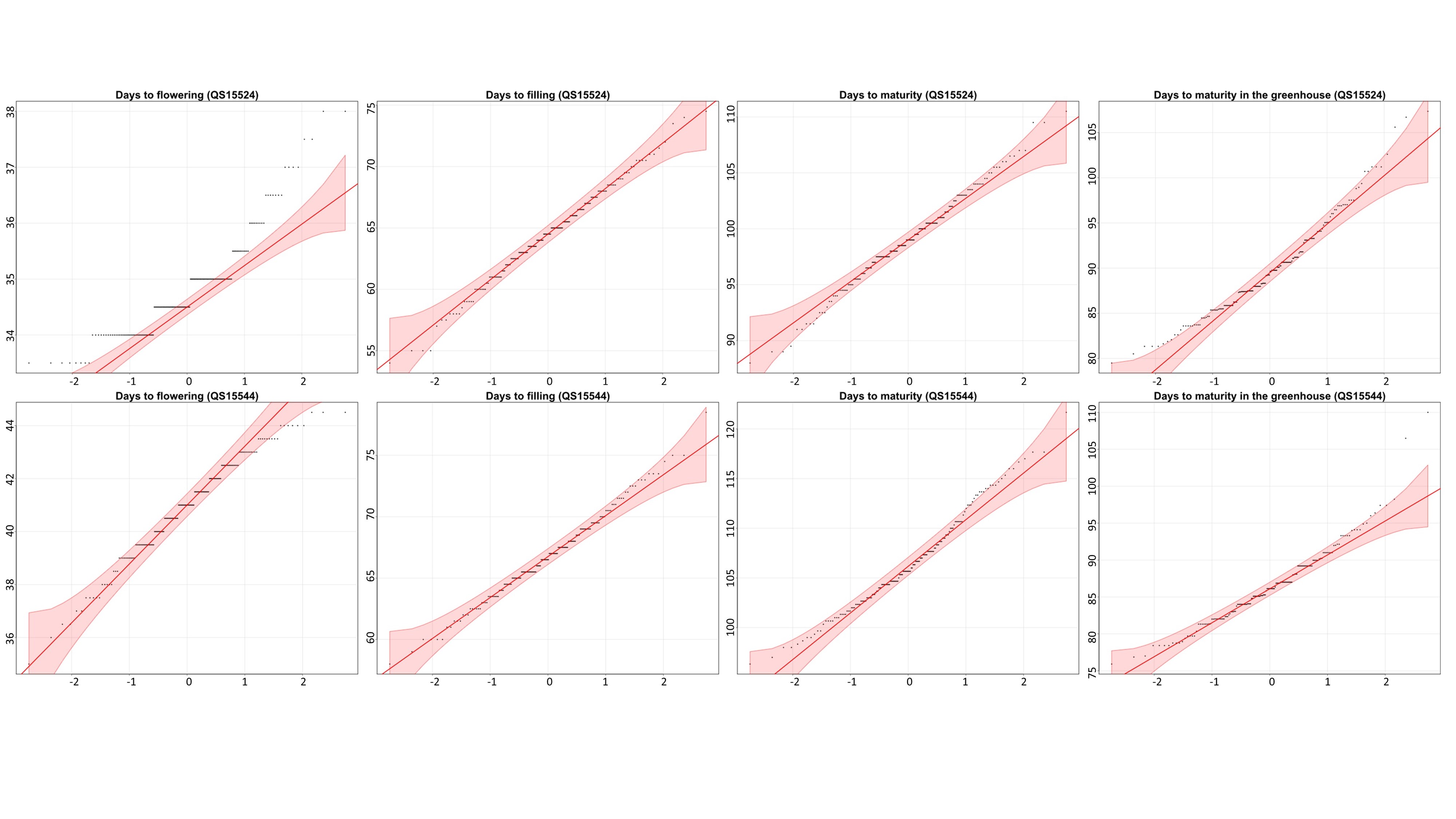

Supplement: Supplementary file 2 [file Image_1.jpeg]
